# An Unusual Case of Acute Confusion in an Elderly Man: Pituitary Apoplexy With Lateral Rectus Palsy

**DOI:** 10.7759/cureus.31064

**Published:** 2022-11-03

**Authors:** Mansoor Zafar, Vivienne Mccallum, Alexandra Nash, Himanshu Kumar, Moniba Waqar, Yaser Mohammed, Cajetan Skowronski, Dua A Malik, Amarah Kiani, Sathis Kumar, Elena Mucci

**Affiliations:** 1 Gastroenterology, General Internal Medicine, Royal Sussex County Hospital, Brighton, GBR; 2 Medicine, Conquest Hospital, St. Leonards-on-Sea, GBR; 3 Family Medicine, Conquest Hospital, St. Leonards-on-Sea, GBR; 4 Internal Medicine, Conquest Hospital, St. Leonards-on-Sea, GBR; 5 Radiology, Conquest Hospital, St. Leonards-on-Sea, GBR; 6 Diabetes and Endocrinology, Conquest Hospital, St. Leonards-on-Sea, GBR; 7 Geriatrics and Stroke Medicine, Conquest Hospital, St. Leonards-on-Sea, GBR

**Keywords:** lateral rectus palsy, mri brain, abducent nerve, abbreviated mental test score (amts), glasgow coma scale (gcs), pituitary apoplexy

## Abstract

Often, the provisional diagnosis for an elderly patient who arrives at the hospital with confusion is presumed to be delirium stemming from confusion usually caused by an infectious cause. The famous mnemonic PINCH ME signifies the ruling out of pain, infection (that usually has a urinary cause), constipation, dehydration, medication (particularly narcotics), and the environment (factors triggering confusion in a patient with a background of dementia). However, we report a rare case of sudden confusion in an elderly male with no previous history of cognitive impairment. This is the first ever reported case to the best of our knowledge of a patient that presented with sudden confusion, impaired extraocular mobility, and spontaneous cranial hemorrhage that was ultimately determined to be due to a hypothalamic and/or a pituitary cause. It signifies a need for prompt evaluation to arrive at an early diagnosis. Additionally, we hope this case report would serve as a guide to look beyond the current mnemonic of PINCH ME and instead to a new mnemonic of 'PINCH ME HOT' where the latter most mnemonic connotes the need to look at a hypothalamic/pituitary, ocular, or traumatic origin for the delirium.

## Introduction

Apoplexy is defined as 'the sudden loss of the ability to feel or move, normally caused by an injury in the brain' [1]. The incidence of pituitary apoplexy has been reported to range from 1% to 26% with a slight male preponderance in most studies [2]. Ranabir et al. have outlined the precipitating factors for pituitary apoplexy including major surgeries, hypertension, coagulopathies, infection, head injury, or radiation although they clarified most cases occur spontaneously [2]. Pituitary apoplexy in most cases is affiliated with pituitary adenoma associated with hemorrhage or infarction following ischemia and hence a potentially life-threatening event [3]. The non-adenomatous pituitary apoplexy has been reported widely in pregnancy [4]. Findling et al. have reported a case of sub-clinical pituitary apoplexy with a history of increased skin pigmentation and headaches post bilateral adrenalectomy for Cushing's disease associated with increased adrenocorticotropic hormone (ACTH) levels, and pituitary hemorrhagic necrosis from pituitary chromophobe adenoma following transsphenoidal surgery [5].

## Case presentation

**Figure 1:**
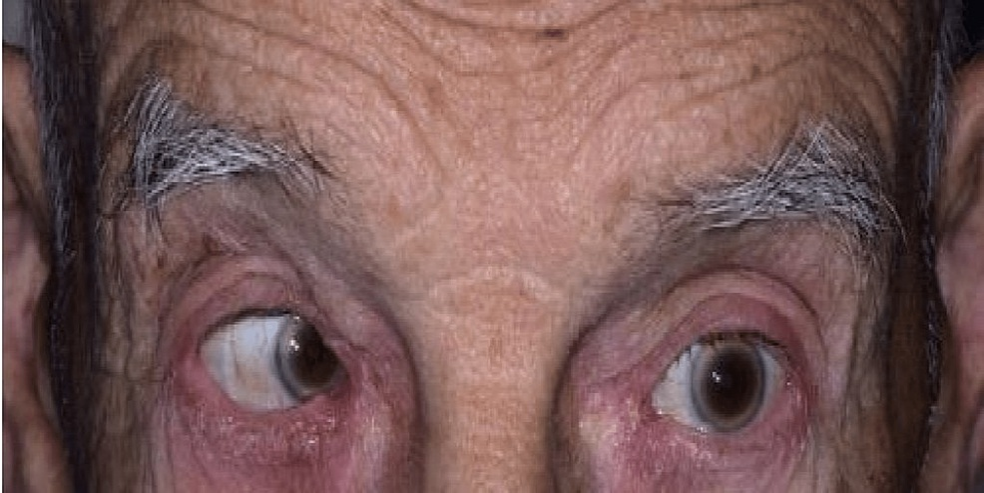
Impaired conjugate lateral deviation with limited left eye abduction with the impression of left abducent (cranial nerve 6) and associated left lateral rectus palsy. Courtesy: Medical illustration department, East Sussex Healthcare NHS Trust

An 86-year-old independently mobile man, with a background history of partial color blindness and benign prostatic hypertrophy (BPH), arrived at the emergency department with a collapse witnessed by his wife and the ambulance crew responding to a 111 call (general health information helpline in the United Kingdom). The wife described that the patient had a two-week history of progressive worsening fatigue, lethargy, nausea, vomiting, acute confusion, and feeling cold. During an assessment on arrival, he scored 4/4 for eye movement (spontaneous eye-opening response), 4/5 for verbal response (confused), and 5/6 for motor response (moves to localized pain) with a cumulative Glasgow coma scale (GCS) of 13/15. However, his abbreviated mental test score (AMTS) was 10/10 and this suggested that there was no dementia upon initial screening at the time of the visit to the hospital. His capillary blood glucose was 6.3 mmol/l. Observations recorded a heart rate of 69/minute, lying blood pressure (BP) of 130/77 mmHg while standing BP with assistance was 121/75 mmHg, respiratory rate of 16/minute, oxygen saturation of 100% on air, with a temperature of 36.3-degree Celsius, and normal skin integrity with no bruises or rashes. Clinical examination did not reveal any abnormality apart from left lateral rectus palsy (Figure 1, Video 1).

**Video 1:** Impaired conjugate lateral deviation. Impression of left abducent (cranial nerve 6) and associated left lateral rectus palsy. Courtesy: Medical illustration department, East Sussex Healthcare NHS Trust

**Figure 2:**
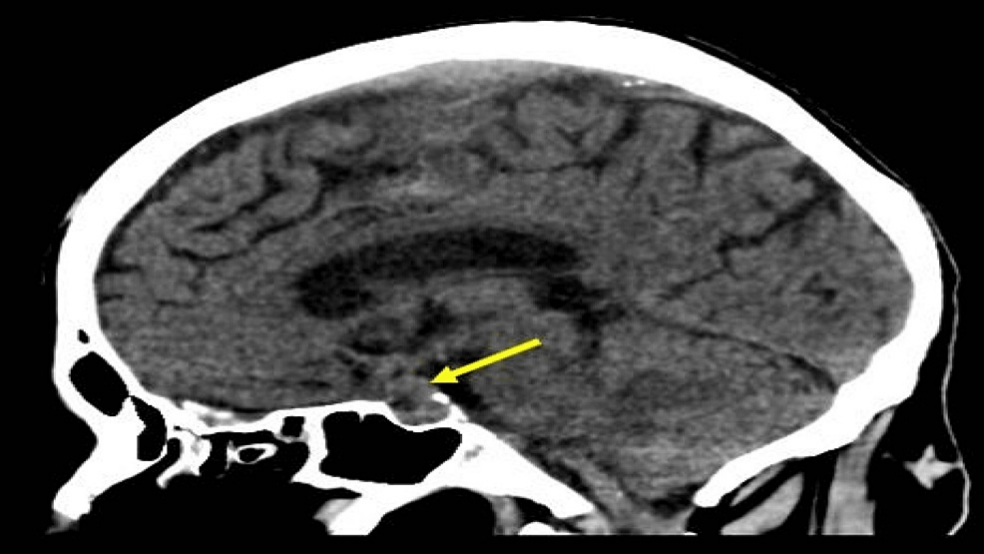
Sagittal non-contrast CT brain shows a well-defined isodense lesion in the pituitary fossa widening of the sella with suprasellar extension (yellow arrow). No frank intracranial hemorrhage was visible.

On inquiry, the patient mentioned that he had been having double vision for the past several years, but he had attributed this to age-related vision changes. The patient had planned to have a review by an optometrist at a later date. An urgent computed tomogram (CT) of the head only showed age-related small vessel ischemic changes. No acute bleeding nor acute ischemic changes were reported (Figure 2).

The chest X-ray (CXR) did not identify any signs of consolidation. His initial blood tests also did not demonstrate any significant abnormality (Table 1).

**Table 1:** Initial blood test results on arrival at the hospital Courtesy: Biochemistry Lab at East Sussex Healthcare NHS Trust

Parameter	Unit of measurement	Reference range	Day 1
Sodium (Na)	mmol/L	133-146	130
Potassium (K)	mmol/L	3.5-5.3	4
Urea	mmol/L	2.5-7.8	9
Creatinine	umol/L	59-104	99
Estimated glomerular filtration rate (eGFR)	ml/min/1.73m^2^	>90	54
C-reactive protein (CRP)	mg/L	up to 5	25
Hemoglobin (Hb)	g/L	130-180	152
White cell count (WCC)	x10^9^/L	4-11	6.9
Neutrophils	x10^9^/L	2-7.5	4.61
Eosinophils	x10^9^/L	0-0.4	0.15
International normalization ratio (INR)	-	0.8-1.2	1.0

Based on the initial assessment and laboratory analysis, a differential diagnosis of stroke or encephalitis with very mild hyponatremia and acute kidney injury (AKI) was made. He was started on intravenous ceftriaxone, acyclovir, and intravenous fluids. The lumbar puncture results showed cerebrospinal fluid (CSF) with a slightly raised protein count of 0.85 grams/litre (reference range: 0.15-0.45 grams/litre), glucose 3.7 millimoles per litre (reference range: 3.3-4.4 mmol/litre) with white cell count (WCC) 12 x106 litre, 100% lymphocytes (reference range: 0-5 x 106 per litre with all lymphocytes with no neutrophils). However, the viral polymerase chain reaction (PCR) on the CSF sample came back negative.

**Figure 3:**
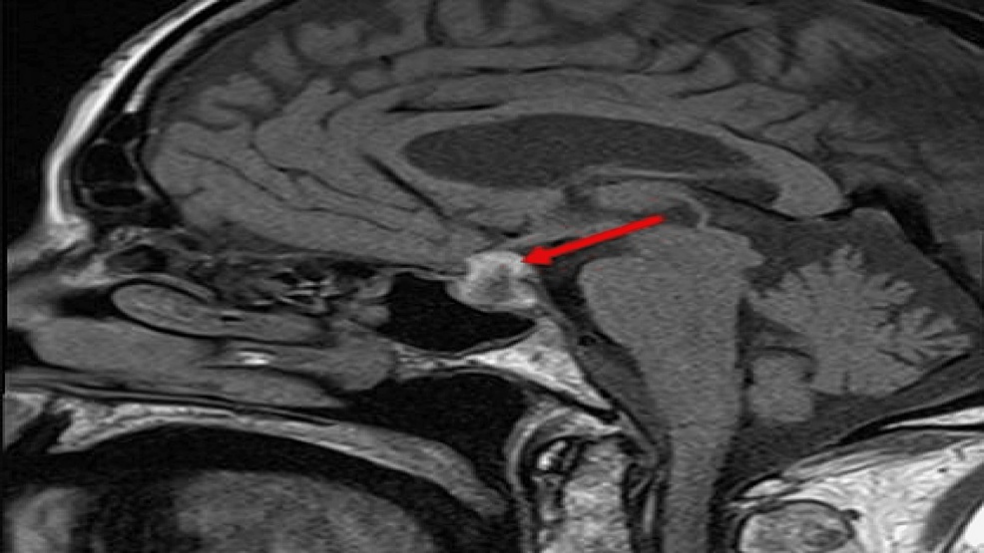
MRI of the brain Sagittal T1 pre-contrast reveals a pituitary region mass with suprasellar extension. It has an intrinsic high T1 signal peripherally, in keeping with blood (red arrow).

The neurology team evaluated the patient and recommended an MRI brain scan be performed. The brain MRI revealed a pituitary mass lesion with suprasellar extension abutting the optic chiasm with evidence of fresh hemorrhage with high T1 signal and susceptibility artifact peripherally within the mass. The appearances were consistent with a diagnosis of pituitary apoplexy (Figure 3).

**Figure 4:**
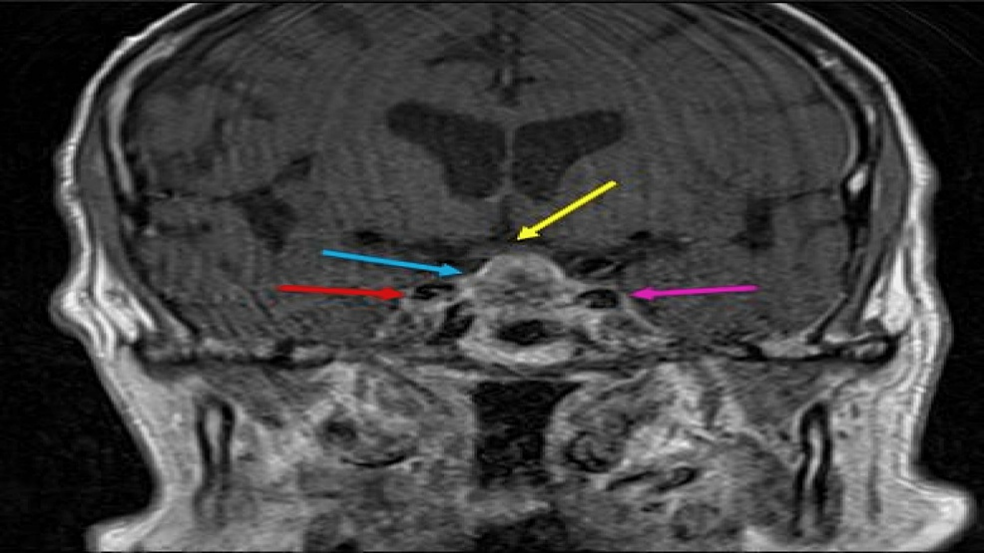
Coronal T1 post-contrast MRI of the pituitary The pituitary mass shows heterogenous enhancement (blue arrow). There is the involvement of the cavernous sinuses (more on the left) with internal carotid arteries along the right side (red arrow) and left side (pink arrow). The suprasellar extension is contacting the undersurface of the optic chiasm (yellow arrow) with no overt compression.

The patient then had a more targeted MRI of the pituitary confirming these findings (Figure 4).

The patient was reviewed by the endocrinology team. The pituitary profile blood test was requested which suggested panhypopituitarism (Table 2).

**Table 2:** Pituitary profile blood tests along with cumulative serum electrolytes * 9 a.m. cortisol ** Reference range plasma ACTH: up to 50 ng/L at 09:00 hours and up to 10 ng/L at midnight

Parameter	Unit of measurement	Reference range	Day 1	Day 7	Day 16
Serum sodium (Na)	mmol/L	133-146	130	129	138
Serum potassium (K)	mmol/L	3.5-5.3	4	3.8	4.4
Serum urea	mmol/L	2.5-7.8	9	7	5.6
Serum creatinine	umol/L	59-104	99	90	89
Estimated glomerular filtration rate (eGFR)	ml/min/1.73m^2^	>90	54	65	76
C-reactive protein (CRP)	mg/L	up to 5	25	17	11
Hemoglobin (Hb)	g/L	130-180	152	147	145
White cell count (WCC)	x10^9^/L	4-11	6.9	5	5.3
Neutrophils	x10^9^/L	2-7.5	4.61	4.5	4.2
Eosinophils	x10^9^/L	0-0.4	0.15	0.2	0.0
International normalization ratio (INR)	-	0.8-1.2	1.0	1.0	1.0
Serum thyroid-stimulating hormone (TSH)	mIU/L	0.27-4.20	0.27	-	-
Serum free thyroxine (T4)	pmol/L	11-22	9	-	-
Serum follicle-stimulating hormone (FSH)	IU/L	1.5-12.4	1.7	-	-
Serum luteinising hormone (LH)	IU/L	1.7-8.6	1.0	-	-
Serum prolactin	mU/l	86-324	24	-	-
Serum cortisol*	nmol/L	137-429	58		-
Serum insulin-like growth factor – 1 (IGF-1)	nmol/L	6.5-24.1	1.2	-	-
Serum testosterone	nmol/L	6.68-25.7	0.26	-	-
Plasma adrenocorticotrophin hormone (ACTH)**	ng/L	up to 46	< 5	-	-

**Figure 5:**
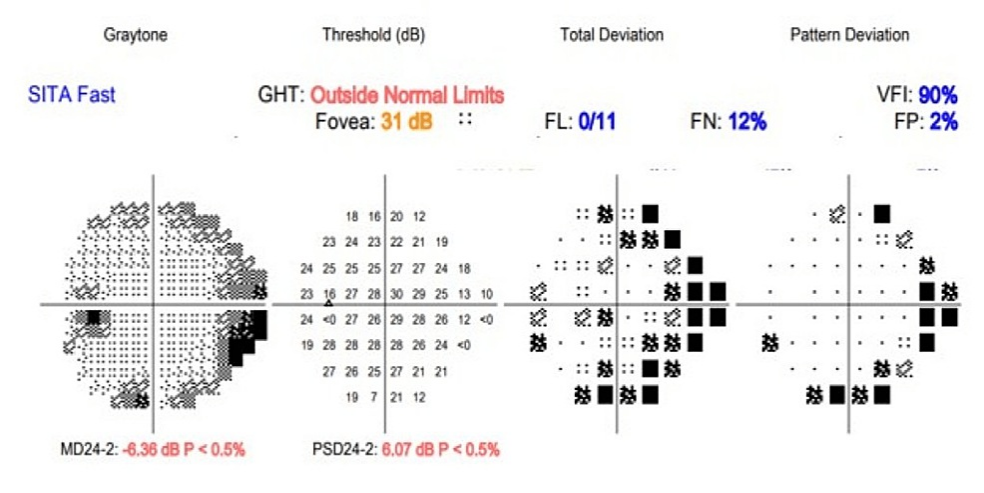
Left eye Humphrey visual field (HVF) analysis chart showing a few more points missed nasally in the left eye SITA: Swedish interactive thresholding algorithm, GHT: Glaucoma hemifield test, FL: Fixation loss, FN: False negative, FP: False positive, VFI: Visual field index, MD: Mean deviation, PSD: Pattern standard deviation

**Figure 6:**
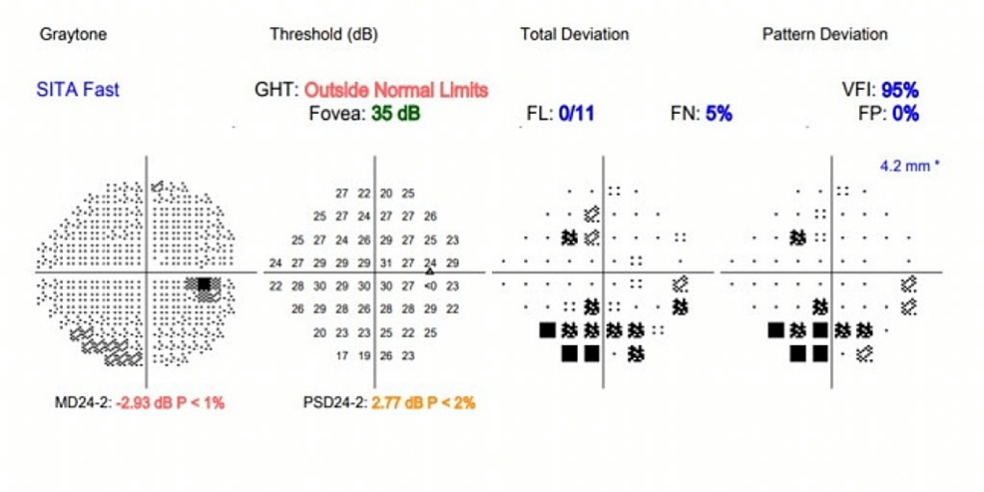
Right eye Humphrey visual field (HVF) analysis chart with comparative lesser affliction. SITA: Swedish interactive thresholding algorithm, GHT: Glaucoma hemifield test, FL: Fixation loss, FN: False negative, FP: False positive, VFI: Visual field index, MD: Mean deviation, PSD: Pattern standard deviation

The patient was commenced on high dose hydrocortisone 50 milligrams (mg) to be taken four times daily for 48 hours then titrated down to a steady dose of hydrocortisone 10 mg in the morning, 5 mg at lunchtime and 5 mg in the evening along with levothyroxine 50 micrograms once a day (OD) until reviewed at the endocrinology clinic. He was also reviewed by the ophthalmology team who confirmed left-sided sixth (abducent nerve) cranial nerve palsy. Humphrey visual field (HVF) analysis showed no defect with exception of a few points missed nasally in the left eye (Figures 5, 6).

The patient felt much better after just 24 hours of steroid treatment and continued to improve. His case was discussed with the neurosurgery team at a tertiary center with advice to continue medical management with outpatient follow-up by the endocrinology team. He was discharged home 16 days after admission. Outpatient follow-up appointments were arranged with endocrinology, ophthalmology, and neurosurgical teams.

The patient was advised not to drive until further notification from the driving and vehicle licensing authority (DVLA). The general practitioner (GP) was advised to arrange for a purple card (to provide support for safe scouting and guidance in the procedures for dealing with an emergency as the patient was on steroids) [6], with periodic blood test analysis in the community. Two weeks after the discharge he was reviewed by the orthoptist team who prescribed prism-corrected spectacles that significantly improved misalignment and resolved diplopia. 

## Discussion

Common clinical symptoms reported to date associated with pituitary apoplexy include headache, nausea and/or vomiting, decreased visual acuity, variable visual field defect, ophthalmoplegia, altered consciousness, pyrexia, and meningeal signs [7]. Rolih et al. have tabulated anatomical localization with clinical presentation in patients with pituitary tumor expansion and/or apoplexy (Table 3) [8].

**Table 3:** Anatomic structures compressed in the vicinity of the pituitary, direction of progress, and the associated clinical symptoms and signs. Source: Modified from Ranabir et al. [2], and Rolih et al. [8] CN I: Cranial nerve I, ophthalmic nerve; CN II: Cranial nerve II, optic nerve; CN IV: Cranial nerve IV, trochlear nerve; CN VI: Cranial nerve VI, abducent nerve; CSF: Cerebrospinal fluid; CN V: Cranial nerve V, trigeminal nerve

Anatomic structures compressed in the vicinity of the pituitary	Direction of tumor expansion and/or progression	Signs and symptoms of compression
Olfactory nerve (CN I)	upwards	Anosmia
Hypothalamus	upwards	Autonomic dysfunction Autonomic dysregulation
Optic pathways (CN II)	upwards	Visual field defects Blindness
Trochlear nerve (CN IV) and Abducent nerve (CN VI)	laterally	Ptosis Pupillary defect ophthalmoplegia
Cavernous sinus	laterally	Epistaxis CSF rhinorrhea
Internal carotid artery (ICA)	laterally	Hemiplegia altered level of consciousness
Trigeminal nerve (CN V)	laterally	Facial pain Corneal anesthesia
Sphenoidal sinus	downward	Epistaxis CSF rhinorrhea

Brain CT can demonstrate sub-arachnoid hemorrhage [9]. However, a cerebral bleed may be missed in CT and may require additional serial CT scans [10]. Special sequence MRI is reported to be more useful than CT scans in characterizing the lesion appropriately [11]. An MRI is useful in the localization and differentiation of tumors and surrounding structures [11]. Dermoid cysts, craniopharyngiomas, lipomas, metastatic melanomas, or any other hemorrhagic tumors are detected with a T1 hyperintense signal [12]. The best advantage of MRI is its usefulness to predict histopathology outcomes in a great majority of patients with pituitary apoplexy [13].

The management of patients with pituitary apoplexy centers around the triad of immediate fluid resuscitation, correction of electrolytes, and steroid replacement. Acute adrenal insufficiency secondary to pituitary apoplexy is reported in two-thirds of patients and is potentially associated with significant mortality and/or morbidity [14]. Hydrocortisone administration up to 100 mg to 200 mg intravenously as a bolus, followed by either continuous infusion of 2 mg to 4 mg/hour or intramuscular administration of 50 mg to 100 mg six hourly (four times a day) is recommended [2]. Other reports of continuous infusion have been recommended due to the saturation kinetics of cortisol-binding globulin [2,15]. Irrespective of the management of the acute phase, following recovery from the acute episode, the recommendations are for quick tapering of the hydrocortisone dose to a maintenance dose of 20 mg to 30 mg per day orally [2,4]. Additional recommendations are for ACTH reserve reassessment in two to three months after the crisis has been resolved [2,4].

The biggest challenge in treating a patient with pituitary apoplexy is involving the specialist teams of endocrinology and neurosurgery as early as possible so that appropriate medical management is initiated with steroids and then proceed to identify patients who would require urgent neurosurgical intervention [2]. The consensus is for patients with hemodynamic compromise and/or worsening eye symptoms from chiasmal compression would require surgical decompression [2,16].

Significant improvement following an early surgical decompression has been reported in patients rendered blind following pituitary apoplexy [6]. However, there are reports of patients with better visual outcomes who were managed conservatively [17,18]. The need for long-term hormone replacement post pituitary apoplexy has been reported to be 40% to 80% for corticosteroids [2,7], 50% to 70% for thyroid hormone [2,7], 6% to 25% for desmopressin [2,7], 40% to 80% for sex steroids, and up to 16% for growth hormone deficiency [2].

The recommendation post four to six weeks of pituitary apoplexy event is to assess pituitary functioning with repeat blood tests for free thyroxine (T4), thyroid-stimulating hormone (TSH), luteinizing hormone (LH), follicle-stimulating hormone (FSH), testosterone in men, oestradiol in women, prolactin (PRL), insulin-like growth factor 1 (IGF1), 9 a.m. cortisol, and growth hormone (GH) [15]. This reassessment of the pituitary-adrenal axis would aid to decide the further long-term requirement of steroid replacement. 

Additionally, there is a need for periodic visual acuity, eye movements, and visual field assessment followed by an annual biochemical repeat of pituitary functions [15]. The Society for Endocrinology Emergency Guidance, United Kingdom, recommends surveillance MRI scans to be performed three to six months post-event followed by an annual MRI for the next five years, followed by two yearly scans [14]. Lastly, annual endocrinology and neurosurgical clinic review are recommended [15].

Lastly, the British Geriatrics Society has a famous mnemonic for delirium (confusion): P-I-N-C-H M-E [19], where P stands for pain, I for infection, N for nutrition, C for constipation, H for hydration, M for medications, and E for environment. We recommend the addition of ‘HOT’ where the H stands for hypothalamic/pituitary, O for ocular, and T for traumatic including post-surgical trauma or spontaneous bleeding as in our patient. Hence, we recommend the new mnemonic of PINCH ME HOT.

## Conclusions

The possibility of pituitary apoplexy should be considered in any patient presenting to the hospital with severe headache and neuro-ophthalmic signs (third, fourth, and sixth cranial nerve palsy). Once subarachnoid hemorrhage (SAH) and meningitis is excluded, patients should be treated with steroids to prevent secondary adrenal insufficiency and help to reduce pressure symptoms.

An MRI brain is the investigation of choice and is preferred over a CT brain to accurately diagnose pituitary apoplexy. It is important to recognize the utilization of imaging such as CT and MRI in arriving at the final diagnosis, and therefore essential for prompt initiation of management as per the Society for Endocrinology Emergency Guidance.

Acute confusion in older adults is often due to delirium commonly precipitated by an infection. We report a rare case of acute confusion in an elderly man due to pituitary apoplexy and recommend a new mnemonic-PINCH ME HOT-to aid prompt evaluation of a patient who arrives at the hospital with confusion.
